# The Blood

**Published:** 1888-12-01

**Authors:** 


### PHYSIOLOGY AND ITS USES.

*(Continued from p. 110.)*

### THE BLOOD.

IN these days, when scientists and other] inquiring persons are very eager to know exactly what is "Man's place in Nature;" how far he may be akin to the gods on the one side, and how nearly he may be related to the apes, or even the amœba on the other, it is a common practice to compare and contrast the different parts of the human organism with corresponding parts in various animals. It is obvious that we may compare the head of a full-grown man with that of a full-grown horse or elephant, and that certain points of agreement will immediately be apparent, and also certain points of difference. Similarly the hands and the feet may be placed side by side, or the heart, the brain, the liver, the spleen, and other distinct and separate organs. The object of all these comparisons is to demonstrate, as we have said, the kind and degree of actual similarity, and, therefore, of presumed kinship between creatures of every sort to which the man of science has access.

It may be asked, "What is the use of such comparison?" From the standpoint of the ploughman or of the bricklayer it is probably of no use. But from the point of view of him who has curious, inquiring, philosophic eyes, it is of the profoundest use. Whether, however, it is of any use or not, certain people will do this kind of thing. They will often arrive at all sorts of erroneous conclusions, as the result of their examinations and speculations; and it thus becomes incumbent on every man of judgment to inform himself of the actual facts, in order that he may be prepared to throw the light of his sound commonsense on the peculiar conclusions of the specialist. The world of thought and opinion is a kind of cauldron. Every man who thinks he has anything to say worth saying should bring it boldly to the cauldron and throw it in. The seething process which follows may result in the quick evaporation of the watery ideas of the speculator, or it may show in course of time that those ideas are of a solid and permanent character—a clear gain to the knowledge and progress of the race. The physiologist has been throwing his ideas into the cauldron for a long time. The alchemy of comparison, knowledge, and good sense is gradually driving away much of the vapour and leaving a solid residuum of palpable and undoubted gain.

No part of the human organism better lends itself to comparison than the blood corpuscles. There are millions of them. They have a very definite shape. Their behaviour under varying circumstance may almost always be calculated upon. They can be compared with the blood corpuscles of other animals with the greatest ease. For all sorts of animals, except the very lowest, are possessed of blood corpuscles, and in this show an undoubted similarity—we do not say kinship—with man. The frog, for example, who is a rather cold-blooded creature, has, as we have seen, his red blood corpuscles, which in shape and size, at any rate, are very similar to our own.

For purposes of comparison it is always well to have a standard of some kind. In comparing blood corpuscles the shortest and readiest method is to make size and shape the standard. The size of a human blood corpuscle, taking our own blood as the starting point, is about $\frac{1}{3200}$ of an inch in diameter, and about a quarter of that measurement in thickness. These being its dimensions, the corpuscle is in no sense globular in shape. It is more like a plate than a globule; a plate, however, of a very shallow kind, and having both sides alike. Physiologists call it a "bi-concave disc," which has the double advantage of looking very learned and being a very correct description at the same time.

The animals which would be most naturally compared with man are the horse, the dog, and the varieties of monkeys. It is found on comparison that most of these have blood corpuscles similar both in size and shape to those of man. This similarity is well marked in almost all mammiferous animals, that is, animals which suckle their young. There is, however, one exception—the camel. In the camel tribe the blood corpuscles, instead of being circular are elliptical in shape. In other respects they have a general correspondence with the ordinary corpuscles of other mammals.

This circumstance of the shape of the blood corpuscles is one of the differentiating characteristics of mammiferous animals as a class. Birds, which are not far removed from men in certain respects, are very different in others. They have backbones like men, and four limbs like him. Their blood corpuscles are, however, not circular but oval discs. Reptiles and fishes are like birds in this particular; their blood corpuscles too are mostly oval in shape. This is one of many similarities existing between birds and reptiles, and which, with other facts, justifies the joining of birds and reptiles together in one great natural class. To the uninstructed eye few creatures could be further apart than the flying birds and the crawling snakes. But when a careful and detailed comparison is made between the two they are found to have more points of agreement than of divergence, and they are therefore constantly classed together.

The size of the corpuscles in different animals varies much more than the shape. We should naturally expect that the larger the animal the larger the corpuscle, and *vice versâ*; but that is by no means always the case. Other circumstances, probably associated with the æration of the blood, seem to be the determining factors in this respect. Birds, for example, have larger corpuscles than man and other mammals. The naked amphibians, who are related to the birds, have the largest corpuscles of all. As a rule, the quadrupeds have smaller corpuscles than man. But this rule affords no good basis for a speculative generalisation, for one animal, the elephant, has larger corpuscles than man or any other mammal. The goat, on the other hand, has very small corpuscles, and so have certain varieties of deer. It need not be considered that "confusion," to use a Hibernianism, "is the order of the day" in the region of the red blood corpuscles. It is true that up to the present time no law has been formulated which is known to control the size and shape of the blood corpuscles in different animals. But that there is such a law, no intelligent physiologist will doubt. If science can affirm any generalisation at all, she can affirm the "reign of law;" and that that reign is universal and permanently potent need not be for a moment questioned. The words "accident" and "confusion" should have no place in the vocabulary of science.

It may seem to non-scientific readers that physiologists have spent a disproportionate measure of labour upon so exceedingly minute a portion of the human organism as the blood corpuscle. But the relative importance of different parts is not always, or even often, to be measured by their size. The progress of knowledge is not to be gauged by the mere extent of the ground covered, but by the thoroughness with which every part is explored. Aristotle was a man of science, even when compared with the greatest scientific luminaries of our own time. But much of Aristotle's knowledge is now elementary, even to a schoolboy. The blood is recognised as the life. That being so, it cannot but play a profoundly important part in everything that affects life; in health and disease, in growth and decay. Much as we know about it, we do not know nearly enough. The best energies of the physiologist and of the physician cannot be better spent than in patient devotion to the study of the normal blood, and to the still wider study of the blood as influenced by food, air, diseases, remedies, poisons, nervous phenomena, and every variety of circumstance that affects the life and wellbeing of man. It is thus, and thus alone, that the true empire of mind over matter can be made real, permanent, and world-wide.

